# Influence of trends in hospital volume over time on patient outcomes for high-risk surgery

**DOI:** 10.1186/s12913-020-05126-4

**Published:** 2020-04-01

**Authors:** Cécile Payet, Stéphanie Polazzi, Jean-Christophe Lifante, Eddy Cotte, Daniel Grinberg, Matthew J. Carty, Stéphane Sanchez, Muriel Rabilloud, Antoine Duclos

**Affiliations:** 1 Health Data Department, Hospices Civils de Lyon, F-69003 Lyon, France; 2 Health Services and Performance Research Lab (HESPER EA7425), Université Claude Bernard Lyon 1, F-69008 Lyon, France; 3 Service de Chirurgie Digestive et Endocrinienne, Hospices Civils de Lyon, Centre Hospitalier Lyon Sud, F-69300 Pierre Bénite, France; 4 Service de Chirurgie Cardio-thoracique et Transplantation, Hôpital Cardio-thoracique Louis Pradel, Lyon-Bron, Avenue du Doyen Lépine, 69500 Bron, France; 5 Brigham and Women’s Hospital, Harvard Medical School, Center for Surgery and Public Health, Boston, MA USA; 6 Hôpitaux Champagne Sud, Centre Hospitalier de Troyes, Pôle Information Médicale Évaluation Performance, Troyes, France; 7 Pôle de Santé Publique, Service de Biostatistique, Hospices Civils de Lyon, Lyon, France; 8 CNRS, UMR5558, Laboratoire de Biométrie et Biologie Evolutive, Equipe Biostatistique-Santé, Villeurbanne, France

**Keywords:** Volume-outcome, Trend, Surgery

## Abstract

**Background:**

The “practice makes perfect” concept considers the more frequent a hospital performs a procedure, the better the outcome of the procedure. We aimed to study this concept by investigating whether patient outcomes improve in hospitals with a significantly increased volume of high-risk surgery over time and whether a learning effect existed at the individual hospital level.

**Methods:**

We included all patients who underwent one of 10 digestive, cardiovascular and orthopaedic procedures between 2010 and 2014 from the French nationwide hospitals database. For each procedure, we identified three groups of hospitals according to volume trend (increased, decreased, or no change). In-hospital mortality, reoperation, and unplanned hospital readmission within 30 days were compared between groups using Cox regressions, taking into account clustering of patients within hospitals and potential confounders. Individual hospital learning effect was investigated by considering the interaction between hospital groups and procedure year.

**Results:**

Over 5 years, 759,928 patients from 694 hospitals were analysed. Patients’ mortality in hospitals with procedure volume increase or decrease over time did not clearly differ from those in hospitals with unchanged volume across the studied procedures (e.g., Hazard Ratios [95%] of 1.04 [0.93–1.17] and 1.08 [0.97–1.21] respectively for colectomy). Furthermore, patient outcomes did not improve or deteriorate in hospitals with increased or decreased volume of procedures over time (e.g., 1.01 [0.95–1.08] and 0.99 [0.92–1.05] respectively for colectomy).

**Conclusions:**

Trend in hospital volume over time did not appear to influence patient outcomes based on real-world data.

**Trial registration:**

NCT02788331, June 2, 2016.

## Background

The relationship between hospital surgical procedures volume and related mortality has been extensively investigated over the past several decades [1]. Numerous studies have reported that patients who undergo operations in hospitals performing a high number of procedures achieve better outcomes [2–10]. Based on these findings, some countries have strived to consolidate specific procedures in high-volume hospitals, with varying results [7, 8, 11, 12]. Consequently, significant methodological flaws regarding the validity of the volume-outcome relationship and the definition of evidence-based volume thresholds have been pointed out [13–17]. Although high-volume hospitals may provide, on average, safer care than low-volume hospitals, some high-volume hospitals may perform poorly while some low-volume hospitals may perform well [18–20]. Nonetheless, hospital-volume continues to be used as a proxy quality metric for high-risk surgeries.

The concept of the volume-outcome relationship is based on the assumption that hospitals that perform a complex procedure more frequently have better outcomes and could manage adverse events more effectively than those who rarely perform the procedure [21, 22]. In the same manner that surgeon or team experience may determine a procedure-specific learning curve [23, 24], this assumption may recapitulate the “practice-makes-perfect” dogma at the institutional level [25, 26]. Most prior studies on volume-outcome relationship have compared outcomes between low- and high-volume hospitals at some point. However, they did not consider the temporal relationship that may exist between volume and outcome under the influence of a dynamic learning effect. Consequently, it remains unclear whether changes in hospital procedural volumes influence patient safety. In this nationwide study, we took another look at the volume-outcome relationship in high-risk procedures by evaluating, at the individual hospital level, the association between trends in volume and patient outcome over time.

## Methods

### Study design and data source

We performed a nationwide observational study to determine whether patient outcomes improve in hospitals with a significantly increased volume of high-risk surgery over time and whether a learning effect existed at the individual hospital level. We first defined three groups of hospitals according to the trend of the volume of surgical procedures over a 5-year period, that is, for a given hospital, the volume of a specific procedure increased, decreased, or did not change. Second, we compared the average patient outcomes and their evolution over time between these three defined hospital groups, taking into account potential confounding factors related to hospital and patient characteristics. To test the robustness of our results, we repeated this scheme across 10 high-risk surgical and interventional procedures in various specialties and considered different patient outcomes. We assumed that if patient outcomes would be influenced by volume change of procedures over time within individual hospitals, those results would be consistent whatever the procedure or outcome studied.

This study used the French Medical Information System (Programme de Médicalisation des Systèmes d’Information [PMSI]), which is a large acute rate hospital database with collected data from all public and private hospitals in France. The database is routinely implemented for the purpose of care reimbursement, which in turn led to strong accuracy and exhaustive collection of data. Thus, no patients were assumed to be lost to follow-up during the study period. Moreover, the PMSI has a system of coding with strict variable definitions and a subset of records audited on a regular basis to avoid coding errors. Inpatient stays are converted into one Diagnosis-Related Group based on standard discharge abstracts containing compulsory information about the patient, primary and secondary diagnoses using the International Classification of Diseases (10th revision - ICD-10 codes), emergency status, and procedural codes associated with the care provided using a detailed classification.

From the PMSI database, we extracted data on patient demographics, co-morbidities according to the Elixhauser algorithm [27], the type and emergency context of the procedure, and discharge by transfer to another acute care hospital. We also characterized each hospital according to its status (i.e., teaching, public, or private for-profit), degree of specialization (i.e., proportion of admissions logged for each studied procedure in the related surgical department), and attraction rate (i.e., the proportion of patients living in another geographical area than that of the hospital location where they underwent each studied procedure). To define patients’ socioeconomic status, we extracted the median income household of patient residence code provided by the National Institute of Statistics and Economic Studies.

This study was approved by Institutional Review Board IRB00009118 (Sud-Est II ethical research committee) and the French Data Protection Authority (CNIL DE-2016-028), and it was registered on clinicaltrial.gov (NCT02788331). In accordance with French ethical directives, the requirement for written informed consent was waived because the study was strictly observational and all data were blinded.

### Study population and outcomes

We included all patients who underwent one of the following 10 procedures from January 1, 2010 to December 31, 2014: resection of a digestive cancer (i.e., colectomy, proctectomy, esophagectomy, gastrectomy, and pancreatectomy), intervention on the cardiovascular system (i.e., percutaneous coronary intervention [PCI]), coronary-artery bypass grafting [CABG], carotid endarterectomy, and elective repair of abdominal aortic aneurysm [AAA]), and urgent hip fracture repair. The choice to focus on those procedures was guided by available evidence suggesting the existence of volume-outcome relationships based on cross-sectional studies [2–5]. Each procedure was identified from the PMSI database by combining specific diagnoses and procedural codes.

For each studied procedure, all patients from hospitals not performing at least one procedure per year were removed from the dataset. Furthermore, patients < 18 years old, who experienced ambulatory care, or with data inaccuracies were excluded. After the washout performed for each procedure separately since 2009, we only selected the first hospitalization of each patient identified as the index stay (except in the case of hip fracture, in which two stays were potentially included if the second stay occurred at least 30 days after the first discharge), using unique, anonymous patient numbers that linked all his/her stays in acute care.

The following patient outcomes were analysed: in-hospital mortality, reoperation, and potentially avoidable hospital readmission. In-hospital mortality and reoperation were defined as death and reoperation, respectively, within a maximum of 30 days post-procedure, whereas potentially avoidable readmission was studied within 30 days of the index stay discharge [28, 29].

### Statistical analysis

To classify hospitals based on their volume change over time, we calculated individual hospital volume for each of the 10 studied procedures as the total number of patients treated by each hospital within each year. Subsequently, hospitals were divided into three groups based on whether their annual procedure volumes were increasing, decreasing or remaining stable over a 5-year period. We used the random slopes of multilevel Poisson models, taking into account the annual repeated measures of hospital volume for each procedure. These slopes were categorized into three groups using the K-means method to avoid arbitrary determination of thresholds and to account for intra-group variances that could vary [30].

Categorical variables were presented using absolute and relative frequencies, and they were compared between groups using the χ2 test. Continuous variables were presented using the means and standard deviation, and they were compared using the Mann-Whitney test. The volume change per year for each hospital was estimated from the random slope of multilevel Poisson model.

For each procedure, to determine if mortality was altered in patients admitted to hospitals with significantly increased volume changes over time and if a learning effect existed at the individual hospital level, we used cox regressions, taking into account the clustering effect of patients within hospitals with robust variance estimator (i.e., patients treated and outcomes within a particular hospital tended to be more similar than those in another hospital), the follow-up that varied from one patient to another, and the hospital discharge that represented a censure of outcome [31, 32]. Furthermore, individual hospital learning effect was investigated by examining the interaction between hospital groups and year of procedure. It was measured using a ratio of hazard-ratio (RHR) comparing the hazard-ratios (HR) between hospitals with increasing or decreasing procedure volume and hospitals with unchanged procedure volume. RHR above 1.0 implied a higher mortality over time in hospitals with increasing or decreasing volume than in hospitals with unchanged volume, while RHR below 1.0 reflected a lower mortality.

To adjust mortality for case mix variations, we considered patient (age, gender, Elixhauser list of comorbidities, type and year of procedure, transfer, emergency admission, and median income) and hospital (hospital status, volume of procedures, specialization degree, and attraction rate) characteristics. Restricted cubic splines were used for continuous variables in the adjustment scheme [33].

To test the robustness of our results, after performing mortality analysis for the 10 procedures, we repeated this approach across secondary outcomes (reoperation and unplanned hospital readmission) using Fine and Gray’s models to consider the, competing risk of death. Model estimates were presented as adjusted hazards ratios with corresponding 95% confidence interval (95% CI). Data manipulation and analyses were performed using SAS version 9.4 (SAS Institute Inc., Cary, NC) and R version 3.2.1 (R Foundation for Statistical Computing, Vienna, Austria) software.

## Results

### Characteristics of patients and hospitals

Over 5 years, 759,928 patients were admitted in 694 French hospitals to undergo one of 10 procedures related to digestive cancer resection, cardiovascular system intervention, or hip fracture repair (Fig. E1). Table 1 shows that not all procedures were performed in all hospitals. The number of hospitals performing each procedure ranged from 45 for CABG to 610 for colectomy. Furthermore, hospital volume varied from one procedure to another, averaging from 29 for gastrectomy to 1269 for PCI.

**Table 1:** Hospital characteristics by procedure

	Hospitals number	Volume of procedures mean (SD)	Status			Specialization degree ^a^ mean (SD)	Attraction rate^b^ mean (SD)
			Teaching	Public or private non-for-profit	Private for profit		
**Colectomy**	610	141.2 (87.5)	30 (4.9%)	319 (52.3%)	261 (42.8%)	5.3 (2.4)	13.8 (14.5)
**Proctectomy**	522	71.0 (54.2)	33 (6.3%)	297 (56.9%)	192 (36.8%)	2.5 (2.3)	16.2 (17.3)
**Esophagectomy**	62	37.0 (58.4)	23 (37.1%)	27 (43.5%)	12 (19.4%)	0.8 (1.1)	33.2 (26.7)
**Gastrectomy**	343	28.5 (22.7)	30 (8.7%)	184 (53.6%)	129 (37.6%)	0.9 (0.9)	16.9 (18.2)
**Pancreatectomy**	166	37.0 (39.0)	28 (16.9%)	83 (50.0%)	55 (33.1%)	0.9 (0.9)	23.5 (22.3)
**PCI**	221	1268.6 (1086.3)	23 (10.4%)	81 (36.7%)	117 (52.9%)	19.6 (10.1)	21.4 (16.5)
**CABG**	45	516.6 (502.2)	22 (48.9%)	18 (40.0%)	5 (11.1%)	7.8 (7.7)	49.3 (21.7)
**AAA repair**	207	77.0 (78.5)	23 (11.1%)	129 (62.3%)	55 (26.6%)	2.1 (1.8)	24.0 (21.2)
**Carotid endarterectomy**	296	215.6 (209.1)	19 (6.4%)	193 (65.2%)	84 (28.4%)	47.7 (26.5)	20.1 (19.6)
**Hip fracture repair**	421	558.5 (437.9)	23 (5.5%)	133 (31.6%)	265 (62.9%)	12.9 (7.9)	11.9 (12.5)

^a^ Proportion of stays for each studied procedure in the surgical department (expressed as a percentage)

^b^ Proportion of patients living in another geographical area that the one of hospital location where they underwent each studied procedure (expressed as a percentage)

PCI percutaneous coronary intervention, CABG coronary-artery bypass grafting, AAA abdominal aortic aneurysm

Total number of patients ranged from 2296 for esophagectomy to 280,369 for PCI (Table 2). Regarding adverse event rates between studied procedures (Table 3), patients who underwent esophagectomy had the highest risk of death (5.4%), reoperation (15.1%) and unplanned hospital readmission (15.7%), while those who underwent carotid endarterectomy had the lowest risk of death (0.9%) and unplanned hospital readmission (5.0%) and those who underwent hip fracture repair had the lowest risk of reoperation (2.5%).

**Table 2:** Patient characteristics by procedure

	Patient number	Men N (%)	Age mean (SD)	No. of different Elixhauser comorbidities^a^ Mean (SD)	Median income, K€ Mean (SD)	Emergency admission N (%)	Discharge by transfer N (%)
**Colectomy**	86,102	44,994 (52.3)	71.6 (12.2)	1.8 (1.6)	20.4 (3.3)	13,201 (15.3)	2771 (3.2)
**Proctectomy**	37,088	22,862 (61.6)	68.5 (11.8)	1.5 (1.5)	20.3 (3.2)	1651 (4.5)	1229 (3.3)
**Esophagectomy**	2296	1858 (80.9)	62.3 (9.5)	2.4 (1.7)	20.0 (3.0)	93 (4.1)	221 (9.6)
**Gastrectomy**	9777	5695 (58.2)	68.8 (12.8)	2.0 (1.7)	20.3 (3.2)	929 (9.5)	500 (5.1)
**Pancreatectomy**	6148	3255 (52.9)	65.0 (11.2)	2.1 (1.7)	20.6 (3.4)	451 (7.3)	316 (5.1)
**PCI**	280,369	209,058 (74.6)	66.1 (13.1)	1.5 (1.4)	20.3 (3.2)	190,154 (67.8)	33,713 (12.0)
**CABG**	23,247	19,019 (81.8)	67.2 (10.4)	2.6 (1.8)	20.0 (2.8)	6586 (28.3)	4927 (21.2)
**AAA repair**	15,935	14,736 (92.5)	74.2 (8.9)	1.8 (1.4)	20.2 (3.3)	0.0 (0.0)	321 (2.0)
**Carotid endarterectomy**	63,829	45,421 (71.2)	72.2 (9.8)	1.7 (1.4)	20.2 (3.3)	4462 (7.0)	1861 (2.9)
**Hip fracture repair**	235,137	60,169 (25.6)	81.6 (11.4)	1.4 (1.4)	20.1 (3.1)	235,137 (100.0)	3713 (1.6)

^a^ Elixhauser comorbidities include congestive heart failure, cardiac arrhythmias, valvular disease, pulmonary circulation disorders, peripheral vascular disorders, hypertension uncomplicated/complicated, paralysis, other neurological disorders, chronic pulmonary disease, diabetes uncomplicated/complicated, hypothyroidism, renal failure, liver disease, peptic ulcer disease excluding bleeding, AIDS/HIV, lymphoma, metastatic cancer, solid tumor without metastasis, rheumatoid arthritis/collagen vascular diseases, coagulopathy, obesity, weight loss, fluid and electrolyte disorders, blood loss anemia, deficiency anemia, alcohol abuse, drug abuse, psychoses, depression

PCI percutaneous coronary intervention, CABG coronary artery bypass grafting, AAA abdominal aortic aneurysm

**Table 3:** Patient outcomes by procedure

	Mortality N (%)	Reoperation N (%)	Unplanned hospital readmission N (%)
**Colectomy**	3271 (3.8)	6349 (7.4)	6432 (7.5)
**Proctectomy**	848 (2.3)	4760 (12.8)	3909 (10.5)
**Esophagectomy**	125 (5.4)	346 (15.1)	360 (15.7)
**Gastrectomy**	451 (4.6)	898 (9.2)	1044 (10.7)
**Pancreatectomy**	283 (4.6)	713 (11.6)	760 (12.4)
**PCI**	6741 (2.4)	41,362 (14.8)	34,074 (12.2)
**CABG**	573 (2.5)	1762 (7.6)	1654 (7.1)
**AAA repair**	204 (1.3)	411 (2.6)	923 (5.8)
**Carotid endarterectomy**	586 (0.9)	1908 (3.0)	3192 (5.0)
**Hip fracture repair**	9059 (3.9)	5827 (2.5)	12,146 (5.2)

*PCI* percutaneous coronary intervention, *CABG* coronary artery bypass grafting, *AAA* abdominal aortic aneurysm

### Relationship between trend in hospital volume and surgical outcomes

**Fig. 1:**
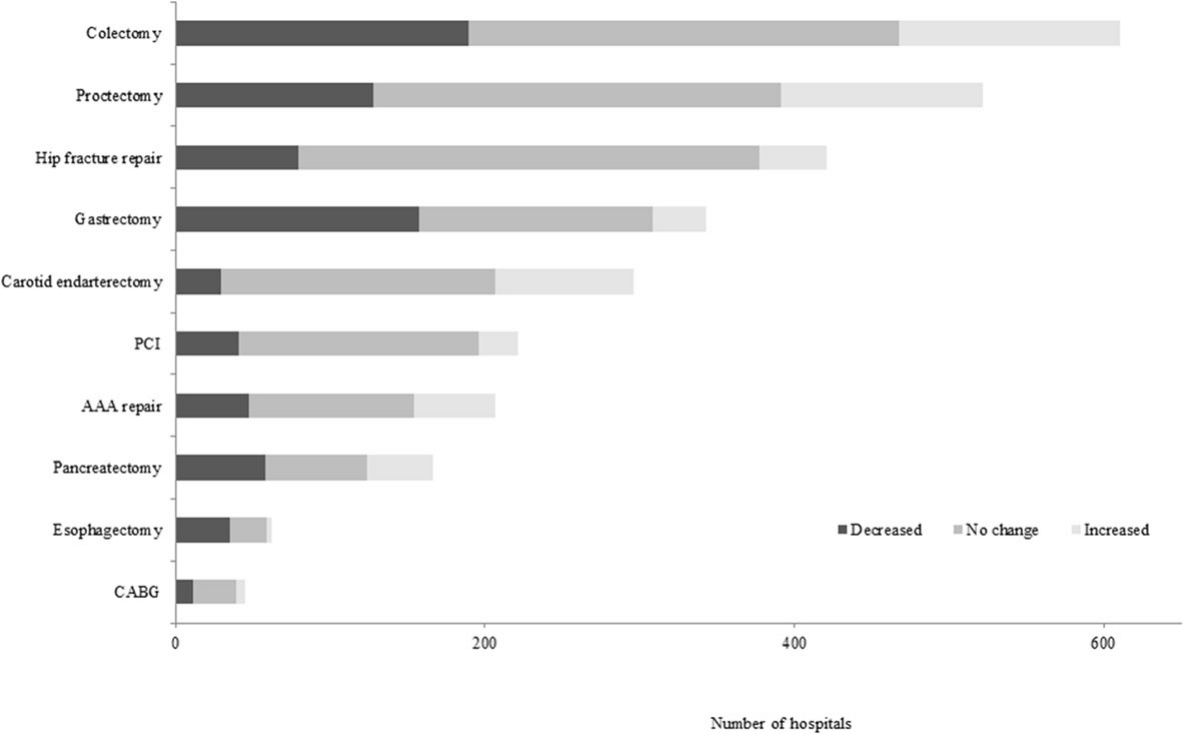
Number of hospitals by trend in volume of procedures between 2010 and 2014. PCI percutaneous coronary intervention, CABG coronary artery bypass grafting, AAA abdominal aortic aneurysm

Figure 1 shows hospital distribution according to the trend in the volume of procedures. The highest proportion of hospitals with unchanged volume was for hip fracture repair (70.8%), while that of hospitals with an increasing volume and those with decreasing volume was for carotid endarterectomy (30.1%) and for esophagectomy (56.4%), respectively. Volume change per year and hospital characteristics according to procedures and trend in volume of procedures are presented in Table E1.

**Fig. 2:**
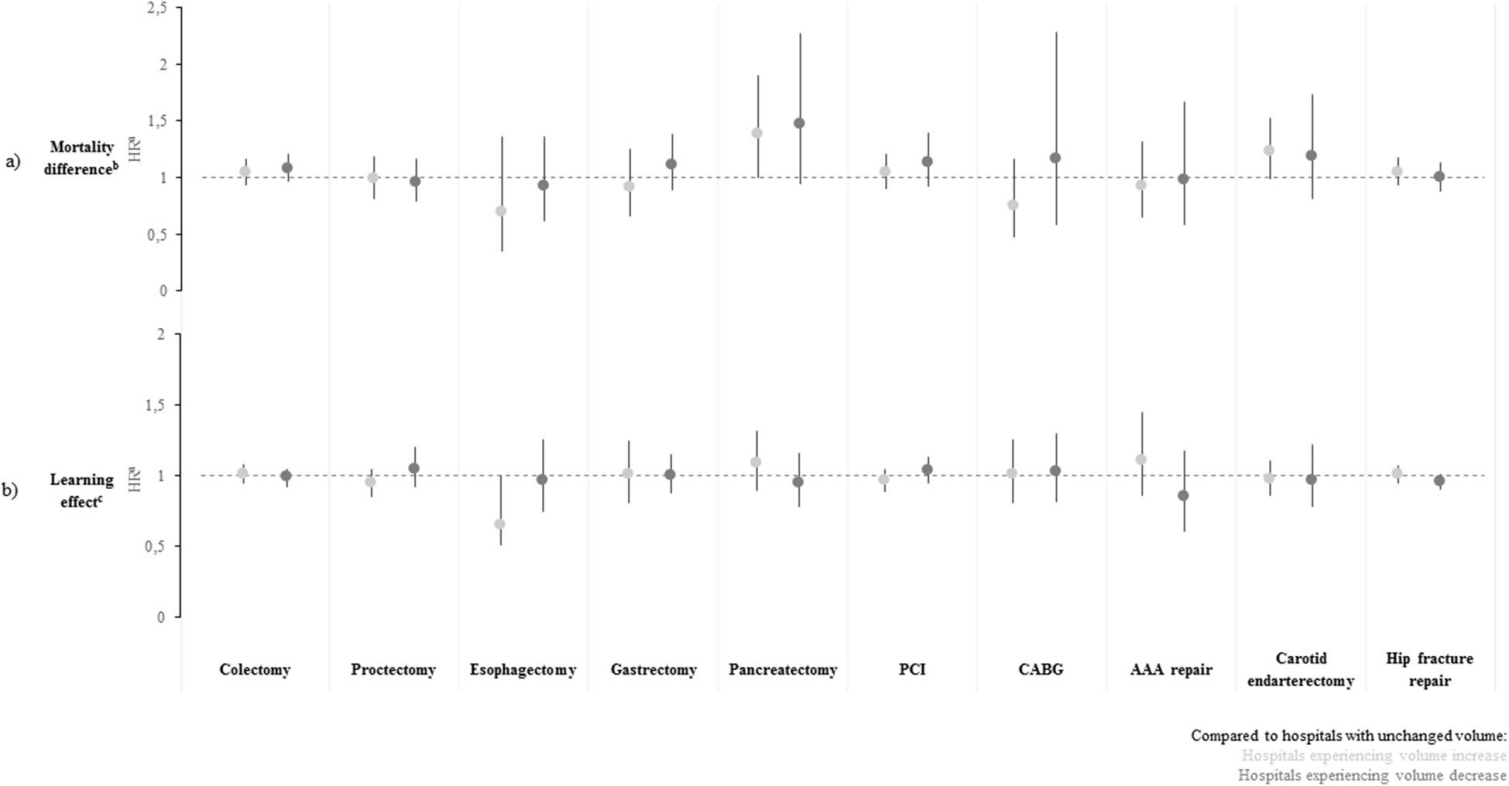
Mortality difference and individual hospital learning effect between hospital groups according to trends in procedures volume from 2010 to 2014. CABG coronary artery bypass grafting, AAA abdominal aortic aneurysm, PCI percutaneous coronary intervention. **a** Comparison of patient mortality across hospitals by comparing hospitals with increasing or decreasing volume with hospitals with unchanged volume. Hazard-ratios estimated from Cox model with adjustment regarding patient (age, gender, Elixhauser list of comorbidities, type and year of procedure, transfer, emergency admission, and median income) and hospital characteristics (hospital status, volume of procedures, specialization degree, and attraction rate). **b** Analyse to determine if mortality improved or deteriorated over time within hospital that increased or decreased its volume. The ratio of hazard ratio (RHR) compared the change in the mortality rate between two groups. A RHR greater than 1 suggests that the increase of mortality over time was greater in hospitals experiencing volume increase/decrease than in hospitals with unchanged volume

Figure 2a (and Table E2) shows that patient mortality were not different among the hospital groups except for pancreatectomy where the mortality rate was higher in hospitals with increasing volume than in hospitals with unchanged volume (HR 95% CI 1.39 [1.02–1.90], *p* = 0.035). Regarding the other outcomes (Fig. E2a and E3a), there was also no difference for most procedures even if some results were inconsistent. Unplanned hospital readmission rate was higher in hospitals with increasing volume for esophagectomy (1.56 [1.08–2.25], *p* = 0.017) and carotid endarterectomy (1.13 [1.01–1.27], *p* = 0.035). Reoperation and unplanned hospital readmission rates were higher in hospitals with decreasing volume (1.31 [1.07–1.61], *p* = 0.010 and 1.34 [1.16–1.56], *p* < 0.001 respectively) for carotid endarterectomy. However, reoperation rate was lower in hospitals with decreasing volume (0.43 [0.22–0.82], *p* = 0.011) for CABG.

Regarding potential individual hospital learning effect (Fig. 2b), no association was found between volume trend and mortality over time except for esophagectomy where mortality decreased over time in hospitals with increasing volume (RHR 0.65 [0.51–0.83], *p* < 0.001). Regarding the other outcomes (Fig. E2b and E3b), there were also no association except for CABG where reoperation rate was lower over time in hospitals with increasing volume (0.71 [0.53–0.94], *p* = 0.018) and higher in those with decreasing volume (1.53 [1.30–1.81], *p* < 0.001), and for AAA repair where it tended to be lower over time in hospitals with decreasing volume for AAA repair (0.78 [0.64–0.96], *p* = 0.018).

## Discussion

We investigated the trends in hospital volume and patient outcomes over time across 10 high-risk procedures. We focused on the “practice-makes-perfect” dogma and assumed that hospitals gain expertise from repeating specific procedure, thereby leading to improved performance.

When comparing outcomes between hospitals that increased, decreased or did not change their volumes, only reoperation and unplanned hospital readmission rates were higher in hospitals with decreasing volume for carotid endarterectomy. Others comparisons revealed either no difference in patient outcomes in hospitals with volume change over time or an inverse relationship to that expected regarding death after pancreatectomy, readmission following esophagectomy or carotid endarterectomy, as well as reoperation after CABG. In the same way, even if individual hospital learning effect may exist regarding death after esophagectomy or reoperation following CABG, patient outcomes for others metrics and procedures did not improve or diminish in hospitals with an increasing or decreasing volume of procedures, respectively. The relationship may even be inverse regarding reoperation after AAA repair. Overall, these results do not support the existence of a robust individual hospital learning effect based on real-world data.

Few prior studies examined the temporal relationship between trends in hospital volume and patient outcomes and they showed contrasting results. These studies involved a limited sample of hospitals and investigated inpatient mortality and/or readmission within 30 days [34–36]. Two studies in trauma centres showed that increasing volume was associated with improving outcomes whereas decreasing volume was associated with worsening outcomes [35, 36]. Another study focused on hip fracture and obtained consistent results with our study, showing that hospitals performing more surgeries over time did not experience outcomes improvement [34]. In our study, to corroborate the “practice-makes-perfect” dogma, we evaluated the consistency of results in a set of distinct procedures. However, we did not find at the hospital level the learning curve that commonly exists for surgeons or surgical teams experiencing improved performance with higher case volume [23, 24]. Several factors may contribute to the relationship between team familiarity and performance, which could prevent the occurrence of intraoperative event. These factors include improved ability to anticipate the actions of other team members, a heightened willingness to relate with one another, and a greater sense of trust [23]. Surgeons who operate together need to continually interpret each other’s cues, both verbal and nonverbal, and to adjust their actions accordingly to stay coordinated. These learning effects refer to human willingness to progress both individually and collectively during the procedure to prevent intraoperative patient morbidity. Furthermore, we endeavoured revealing a potential learning effect at the hospital level through a gradual optimization of surgical care process with case repetition. Accordingly, we not only considered the operating room activities but also studied the ability of hospital systems to prevent postoperative complications or take actions necessary to mitigate untoward consequences. A hospital’s proficiency in minimizing failure to rescue could be related to a variety of factors, such as available resources and perioperative care organization [37]. The quality of recruitment, preoperative evaluation, anaesthetic management, postoperative nursing, patient follow-up, and an integrated care system with enhanced healthcare workers collaboration are also essential for safe surgery.

This study has some potential limitations. Data were extracted from large hospital databases, which have been initially implemented for billing inpatient stays. Thus, motivation of data coders was possibly influenced by financial stakes rather than epidemiological accuracy [38]. Risk adjustment could only account for factors that can be identified and measured accurately from these data [39]. Although we considered hospital and patient characteristics in the adjustment scheme, this may not be sufficient to ascertain the effect of the differential case mix on surgical outcome between groups since they were not randomly assigned. Moreover, we could not adjust patient outcomes for the volume of procedures and learning curve at the individual surgeon level as no data are available. Consequently, the individual hospital learning effect independent of the surgeon’s ability could not be estimated. We also could not identify whether the increase in volume was due to an increase in surgeon number performing the procedure in the hospital or due to an increase in the number of procedures by the same surgeons. Furthermore, specific complications for each procedure, such as major adverse cardiac and cerebrovascular events for CABG or stroke for carotid surgery, were not monitored. To analyse jointly the results across various procedures, we opted for generic outcomes. The unclear link between those outcomes and surgical care quality may explain the difficulties in revealing the existence of a potential individual hospital learning effect. This may also be attributable to the fact that many surgical procedures have been performed for a long time and hospitals may have achieved the benefits of learning well before the study period, precluding our ability to evidence practice-makes-perfect. Hence, individual hospital learning effects would be easier to reveal with relatively new procedures, wherein hospitals initiate several changes in the facilities and staff for safe surgery. Finally, a delay in the effect of the change in hospital volume on patient outcomes is possible. Assuming that hospitals would require a sustained volume increase for several years to produce an observable effect on patient outcomes, we may have failed to identify a learning effect because of the limited duration of follow-up of hospital behaviour.

Our findings have implications for the efforts aimed at surgical care improvement. We studied a wide range of procedures to investigate the dynamics of volume-outcome relationship within hospitals and showed that a significant increase in volume of procedures over time does not necessarily enhance patient safety. Accordingly, temporal variation in the volume of procedures would not seem a valid surrogate of surgical quality for guiding hospitals’ pay-for-performance strategies or providing licensure of surgical practice. Patient outcomes at the individual hospital level would be influenced by factors other than potential learning effect. This may help explain the low mortality [18–20] and the good outcomes [40] in some low-volume hospitals, and possibly some other factors enabled hospitals to achieve excellent outcomes independent of the volume of procedures. For example, higher surgical quality could be attributed to better care organization or the availability of effective medical technologies. Current health policies promote centralizing specific procedures in high-volume referral centres to improve patient outcomes. However, such could result in disparities in patient access to safe surgery, unreasonable travel burdens, and potential delays in operations [41–43]. Hence, in areas where access to referral centres is limited, importing optimal practices may be essential to delivering high-quality surgery. Providing homogeneous surgical quality across the country represents an alternative to centralization, which requires the identification of best systems to achieve excellent outcome and effective strategies to implement these systems from one hospital to another.

## Conclusion

The “practice makes perfect” concept considers the more frequent a hospital performs a procedure, the better the outcome of the procedure. In this study, instead of compared outcomes between low- and high-volume hospitals at some point, we considered the temporal relationship that may exist between volume and outcome under the influence of a dynamic learning effect. Trend in hospital volume over time did not appear influencing consistently patient outcomes across ten high-risk procedures based on real-world data.

## Supplementary information

**Additional file 1: Figure E1.** Study flowchart. **Table E1.** Hospital characteristics by procedure and trend in volume of procedures between 2010 and 2014. **Figure E2.** Reoperation difference and individual hospital learning effect between volume trend groups according to procedures between 2010 and 2014. **Figure E3.** Unplanned hospital readmission difference and individual hospital learning effect between volume trend groups according to procedures between 2010 and 2014. **Table E2.** Outcome difference and individual hospital learning effect between hospital groups according to trends in procedures volume from 2010 to 2014.

## Data Availability

The data that support the findings of this study are available from the French Medical Information System maintained by the Technical Agency for Information on Hospital Care. However, restrictions apply to the availability of these data, which were used under license for the current study, and so are not publicly available. To obtain this dataset for an international researcher: Email: demande_base@atih.sante.fr.

## Abbreviations

PMSI: Programme de Médicalisation des Systèmes d’Information
PCI: Percutaneous coronary intervention
CABG: Coronary-artery bypass grafting
AAA: Elective repair of abdominal aortic aneurysm
RHR: Ratio of hazard-ratio
HR: Hazard-ratios
