# Supplementary material for: Influence of trends in hospital volume over time on patient outcomes for high-risk surgery
Source: BMC Health Serv Res. 2020 Apr 1;20:274. doi: 10.1186/s12913-020-05126-4 (PMC7114802; doi:10.1186/s12913-020-05126-4)
Supplement: Supplementary file 1 — Additional file 1: Figure E1. Study flowchart. Table E1. Hospital characteristics by procedure and trend in volume of procedures between 2010 and 2014. Figure E2. Reoperation difference and individual hospital learning effect between volume trend groups according to procedures between 2010 and 2014. Figure E3. Unplanned hospital readmission difference and individual hospital learning effect between volume trend groups according to procedures between 2010 and 2014. Table E2. Outcome difference and individual hospital learning effect between hospital groups according to trends in procedures volume from 2010 to 2014. [file 12913_2020_5126_MOESM1_ESM.docx]

**Appendix**


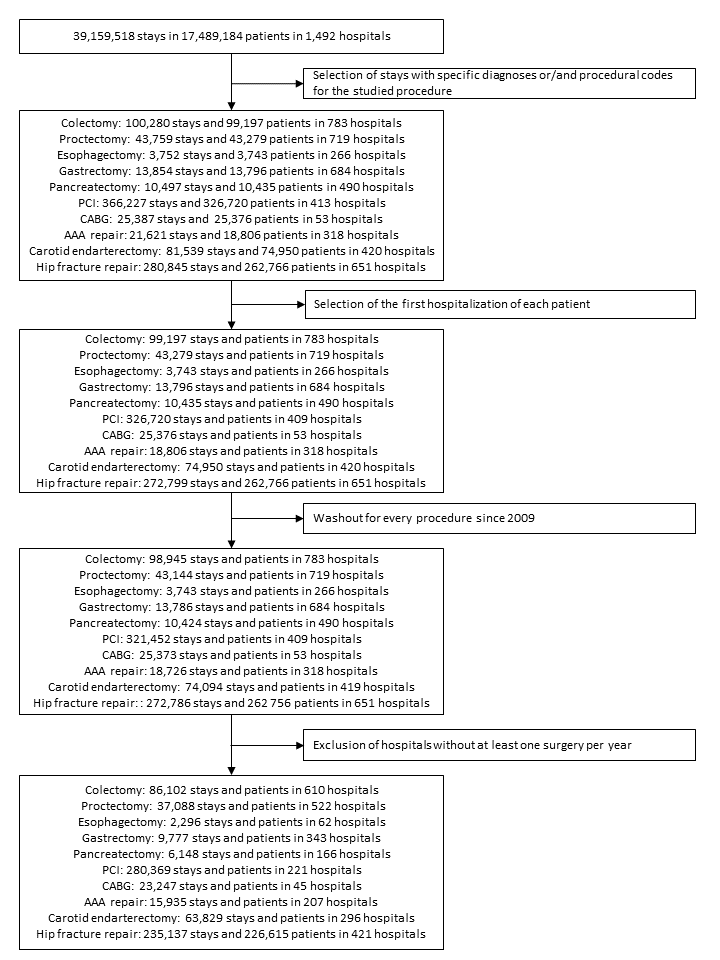


**Figure E1** Study flowchart

**Table E1** Hospital characteristics by procedure and trend in volume of procedures between 2010 and 2014.

|  | |  | **Decreased**  **N (%)** | **No change**  **N (%)** | **Increased**  **N (%)** | **P-value** |
| --- | --- | --- | --- | --- | --- | --- |
| **Colectomy** | | |  |  |  |  |
|  | | Volume change per year, mean (SD) | -4.7 (1.9) | 0.2 (1.4) | 5.4 (2.4) | <0.001 |
|  | | Volume of procedures, mean (SD) | 115.4 (83.9) | 145.2 (86.2) | 167.4 (86.3) | <0.001 |
|  | | Status |  |  |  | 0.136 |
|  | | *Teaching* | 6 (3.2%) | 12 (4.3%) | 12 (8.5%) |  |
|  | | *Private for profite* | 108 (57.1%) | 142 (50.9%) | 69 (48.6%) |  |
|  | | *Public or private non-for-profit* | 75 (39.7%) | 125 (44.8%) | 61 (43.0%) |  |
|  | | Specializaton degree^a^, mean (SD) | 5.0 (2.2) | 5.3 (2.1) | 5.8 (3.0) | 0.020 |
|  | | Attraction rate^b^, mean (SD) | 12.4 (14.1) | 14.6 (15.0) | 14.1 (13.9) | 0.180 |
| **Protectomy** | | |  |  |  |  |
|  | | Volume change per year, mean (SD) | -5.9 (2.4) | -0.7 (1.6) | 5.0 (2.6) | <.001 |
|  | | Volume of procedures, mean (SD) | 66.6 (50.9) | 66.5 (56.1) | 84.5 (51.8) | 0.004 |
|  | | Status |  |  |  | 0.002 |
|  | | *Teaching* | 2 (1.6%) | 19 (7.2%) | 12 (9.2%) |  |
|  | | *Private for profite* | 88 (68.8%) | 133 (50.6%) | 76 (58.0%) |  |
|  | | *Public or private non-for-profit* | 38 (29.7%) | 111 (42.2%) | 43 (32.8%) |  |
|  | | Specializaton degree^a^, mean (SD) | 2.5 (1.9) | 2.5 (2.5) | 2.6 (2.0) | 0.893 |
|  | | Attraction rate^b^, mean (SD) | 14.0 (16.2) | 15.9 (17.3) | 18.9 (18.2) | 0.067 |
| **Esophagectomy** | | |  |  |  |  |
|  | | Volume change per year, mean (SD) | -3.0 (2.8) | 5.4 (3.1) | 20.6 (6.8) | <0.001 |
|  | | Volume of procedures, mean (SD) | 35.8 (67.6) | 31.6 (31.7) | 95.0 (96.3) | 0.206 |
|  | | Status |  |  |  | 0.759 |
|  | | *Teaching* | 11 (31.4%) | 11 (45.8%) | 1 (33.3%) |  |
|  | | *Private for profite* | 16 (45.7%) | 9 (37.5%) | 2 (66.7%) |  |
|  | | *Public or private non-for-profit* | 8 (22.9%) | 4 (16.7%) | 0 (0.0%) |  |
|  | | Specializaton degree^a^, mean (SD) | 0.6 (0.7) | 1.0 (1.6) | 1.1 (0.9) | 0.479 |
|  | | Attraction rate^b^, mean (SD) | 28.0 (23.1) | 40.3 (30.1) | 36.9 (34.9) | 0.222 |
| **Gastrectomy** | | |  |  |  |  |
|  | | Volume change per year, mean (SD) | 2.4 (0.9) | 0.4 (0.5) | -1.0 (0.6) | <0.001 |
|  | | Volume of procedures, mean (SD) | 49.0 (27.5) | 30.0 (23.0) | 22.5 (18.1) | <0.001 |
|  | | Status |  |  |  | 0.011 |
|  | | *Teaching* | 8 (22.9%) | 15 (9.9%) | 7 (4.5%) |  |
|  | | *Private for profite* | 15 (42.9%) | 78 (51.7%) | 91 (58.0%) |  |
|  | | *Public or private non-for-profit* | 12 (34.3%) | 58 (38.4%) | 59 (37.6%) |  |
|  | | Specializaton degree^a^, mean (SD) | 1.2 (1.0) | 1.0 (1.1) | 0.7 (0.4) | 0.001 |
|  | | Attraction rate^b^, mean (SD) | 23.0 (17.7) | 17.4 (18.9) | 15.0 (17.3) | 0.053 |
| **Pancreatectomy** | | |  |  |  |  |
|  | | Volume change per year, mean (SD) | -4.5 (2.0) | 2.2 (1.9) | 9.3 (3.4) | <0.001 |
|  | | Volume of procedures, mean (SD) | 22.4 (29.7) | 39.6 (35.0) | 53.2 (48.8) | <0.001 |
|  | | Status |  |  |  | 0.010 |
|  | | *Teaching* | 3 (5.2%) | 12 (18.2%) | 13 (31.0%) |  |
|  | | *Private for profite* | 35 (60.3%) | 33 (50.0%) | 15 (35.7%) |  |
|  | | *Public or private non-for-profit* | 20 (34.5%) | 21 (31.8%) | 14 (33.3%) |  |
|  | | Specializaton degree^a^, mean (SD) | 0.6 (0.4) | 1.0 (0.8) | 1.3 (1.3) | <0.001 |
|  | | Attraction rate^b^, mean (SD) | 19.1 (22.2) | 24.8 (22.0) | 27.7 (22.2) | 0.140 |
| **PCI** | | |  |  |  |  |
|  | | Volume change per year, mean (SD) | -15.4 (8.5) | 2.1 (4.9) | 24.8 (16.6) | <0.001 |
|  | | Volume of procedures, mean (SD) | 741.8 (860.2) | 1491.8 (1117.8) | 748.9 (699.5) | <0.001 |
|  | | Status |  |  |  | 0.085 |
|  | | *Teaching* | 1 (2.4%) | 21 (13.5%) | 1 (4.0%) |  |
|  | | *Private for profite* | 20 (48.8%) | 54 (34.8%) | 7 (28.0%) |  |
|  | | *Public or private non-for-profit* | 20 (48.8%) | 80 (51.6%) | 17 (68.0%) |  |
|  | | Specializaton degree^a^, mean (SD) | 14.0 (7.8) | 21.3 (10.2) | 18.6 (9.4) | <0.001 |
|  | | Attraction rate^b^, mean (SD) | 21.7 (18.5) | 22.0 (16.0) | 17.4 (16.2) | 0.427 |
| **CABG** | | |  |  |  |  |
|  | | Volume change per year, mean (SD) | -40.2 (11.0) | -3.3 (9.0) | 29.5 (6.5) | <0.001 |
|  | | Volume of procedures, mean (SD) | 193.6 (109.9) | 683.3 (545.7) | 330.8 (401.0) | 0.011 |
|  | | Status |  |  |  | 0.033 |
|  | | *Teaching* | 2 (18.2%) | 18 (64.3%) | 2 (33.3%) |  |
|  | | *Private for profite* | 6 (54.5%) | 9 (32.1%) | 3 (50.0%) |  |
|  | | *Public or private non-for-profit* | 3 (27.3%) | 1 (3.6%) | 1 (16.7%) |  |
|  | | Specializaton degree^a^, mean (SD) | 3.6 (2.6) | 10.0 (8.5) | 5.6 (7.1) | 0.047 |
|  | | Attraction rate^b^, mean (SD) | 50.4 (27.0) | 48.7 (19.6) | 50.2 (24.7) | 0.972 |
| **AAA repair** | | |  |  |  |  |
|  | | Volume change per year, mean (SD) | -10.5 (5.5) | 1.9 (3.7) | 15.2 (5.3) | <0.001 |
|  | | Volume of procedures, mean (SD) | 64.3 (101.2) | 72.8 (73.6) | 96.6 (61.4) | 0.089 |
|  | | Status |  |  |  | 0.703 |
|  | | *Teaching* | 5 (10.6%) | 10 (9.3%) | 8 (15.1%) |  |
|  | | *Private for profite* | 32 (68.1%) | 66 (61.7%) | 31 (58.5%) |  |
|  | | *Public or private non-for-profit* | 10 (21.3%) | 31 (29.0%) | 14 (26.4%) |  |
|  | | Specializaton degree^a^, mean (SD) | 2.4 (1.7) | 1.9 (1.7) | 2.2 (2.1) | 0.275 |
|  | | Attraction rate^b^, mean (SD) | 22.7 (18.9) | 21.1 (20.5) | 31.2 (23.2) | 0.016 |
| **Carotid endarterectomy** | | |  |  |  |  |
|  | Volume change rate per year, mean (SD) | | -15.9 (6.0) | -2.6 (3.3) | 8.8 (5.4) | <0.001 |
|  | Volume of procedures, mean (SD) | | 136.2 (153.7) | 243.4 (233.9) | 186.0 (156.8) | 0.010 |
|  | Status | |  |  |  | 0.179 |
|  | *Teaching* | | 0 (0.0%) | 11 (6.2%) | 8 (9.0%) |  |
|  | *Private for profite* | | 21 (72.4%) | 122 (68.5%) | 50 (56.2%) |  |
|  | *Public or private non-for-profit* | | 8 (27.6%) | 45 (25.3%) | 31 (34.8%) |  |
|  | Specializaton degree^a^, mean (SD) | | 53.0 (26.1) | 48.0 (28.1) | 45.3 (23.1) | 0.386 |
|  | Attraction rate^b^, mean (SD) | | 20.5 (21.9) | 20.9 (19.9) | 18.1 (18.3) | 0.536 |
| **Hip Fracture repair** | | |  |  |  |  |
|  | | Volume change per year, mean (SD) | -12.1 (10.4) | 3.6 (4.7) | 30.7 (14.4) | <0.001 |
|  | | Volume of procedures, mean (SD) | 330.3 (413.3) | 640.6 (432.0) | 412.3 (331.9) | <0.001 |
|  | | Status |  |  |  | <0.001 |
|  | | *Teaching* | 5 (6.3%) | 16 (5.4%) | 2 (4.5%) |  |
|  | | *Private for profite* | 43 (54.4%) | 76 (25.5%) | 14 (31.8%) |  |
|  | | *Public or private non-for-profit* | 31 (39.2%) | 206 (69.1%) | 28 (63.6%) |  |
|  | | Specializaton degree^a^, mean (SD) | 8.8 (8.0) | 14.6 (7.5) | 8.8 (6.3) | <0.001 |
|  | | Attraction rate^b^, mean (SD) | 12.0 (13.0) | 12.1 (12.8) | 10.0 (9.3) | 0.583 |

^a^ Proportion of stays for each studied procedure in the surgical department (expressed as a percentage)

^b^ Proportion of patients living in another geographical area that the one of hospital location where they underwent each studied procedure (expressed as a percentage)

PCI percutaneous coronary intervention, CABG coronary-artery bypass grafting, AAA abdominal aortic aneurysm


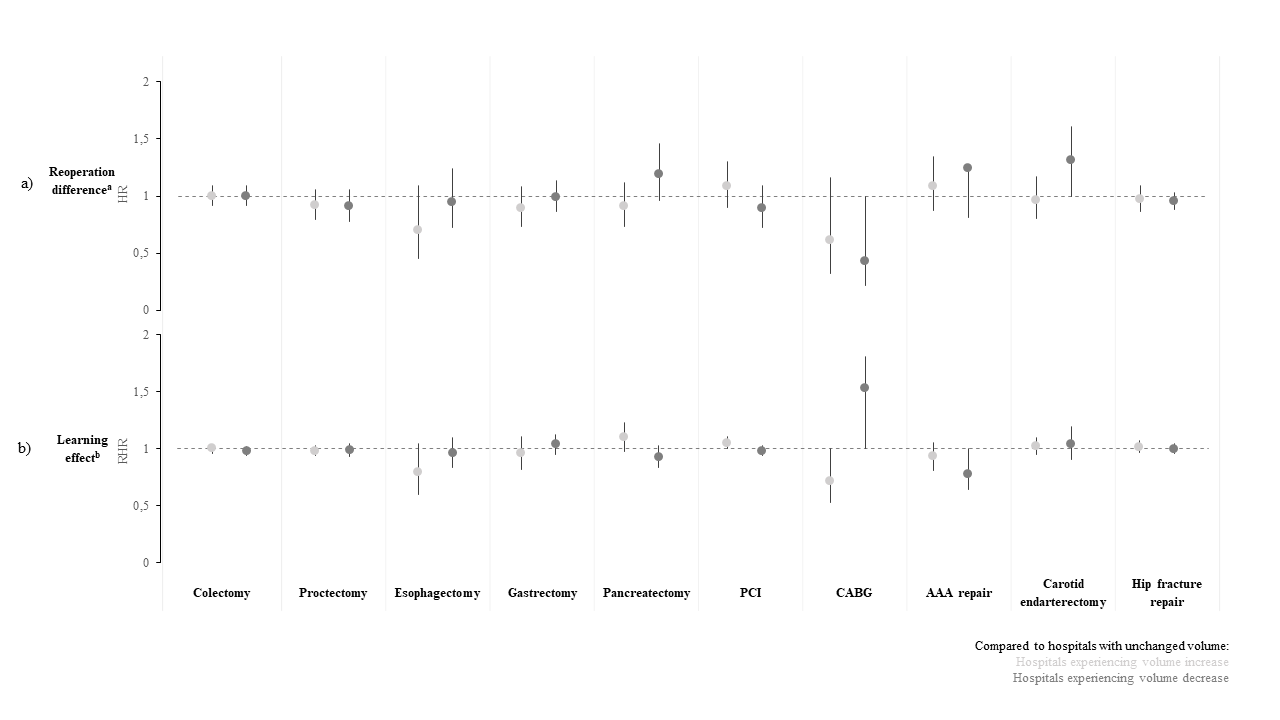


**Figure E2** Reoperation difference and individual hospital learning effect between volume trend groups according to procedures. between 2010 and 2014. CABG coronary artery bypass grafting. AAA abdominal aortic aneurysm. PCI percutaneous coronary intervention. ^a^ Comparison of patient reoperation across hospitals by comparing hospitals with increasing or decreasing volume with hospitals with unchanged volume. Hazard-ratios estimated from Fine and Gray’s competing risk model considering patient characteristics (age. gender. Elixhauser list of comorbidities. type and year of procedure. transfer. emergency admission. and median income) and hospital characteristics (hospital status. volume of procedures. specialization degree. and attraction rate). The clustering effect of patients within hospitals was taken into account with robust variance estimator.

^b^ Analyse to determine if reoperation improved or deteriorated over time within hospital that increased or decreased its volume. The ratio of hazard ratio (RHR) compare the change in the reoperation rate between two groups. A RHR greater than 1 suggests that the increase of reoperation over time was greater in hospitals experiencing volume increase/decrease than in hospitals with unchanged volume


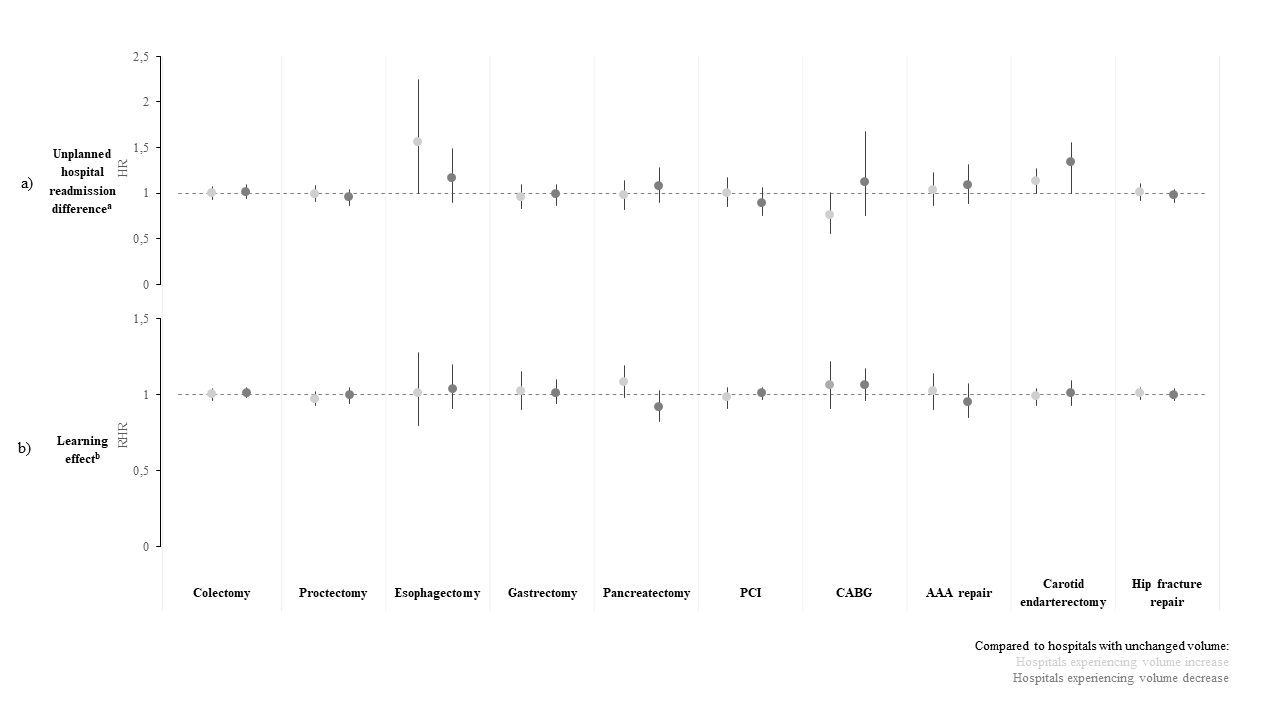


**Figure E3** Unplanned hospital readmission difference and individual hospital learning effect between volume trend groups according to procedures. between 2010 and 2014.

CABG coronary artery bypass grafting. AAA abdominal aortic aneurysm. PCI percutaneous coronary intervention. ^a^ Hazard-ratios estimated from Fine and Gray’s competing risk model considering patient characteristics (age. gender. Elixhauser list of comorbidities. type and year of procedure. transfer. emergency admission. and median income) and hospital characteristics (hospital status. volume of procedures. specialization degree. and attraction rate). The clustering effect of patients within hospitals was taken into account with robust variance estimator. ^b^ Comparison of patient unplanned hospital readmission across hospitals by comparing hospitals with increasing or decreasing volume with hospitals with unchanged volume. ^c^ Analyse to determine if unplanned hospital readmission improved or deteriorated over time within hospital that increased or decreased its volume. The ratio of hazard ratio (RHR) compare the change in the unplanned readmission rate between two groups. A RHR greater than 1 suggests that the increase of readmission over time was greater in hospitals experiencing volume increase/decrease than in hospitals with unchanged volume.

Table E2 – Outcome difference and individual hospital learning effect between hospital groups according to trends in procedures volume from 2010 to 2014

|  | **Mortality** | | **Reoperation** | | **Readmission** | |
| --- | --- | --- | --- | --- | --- | --- |
|  | **Difference**  **HR [95%CI]** | **Learning effect**  **HR [95%CI]** | **Difference**  **HR [95%CI]** | **Learning effect**  **HR [95%CI]** | **Difference**  **HR [95%CI]** | **Learning effect**  **HR [95%CI]** |
| **Colectomy** *(ref = Unchanged volume)* | |  |  |  |  |  |
| *Increased* | 1.04 [0.93 1.17] | 1.01 [0.95 1.08] | 1.00 [0.92 1.09] | 1.00 [0.96 1.03] | 1.00 [0.93 1.07] | 1.00 [0.96 1.04] |
| *Decreased* | 1.08 [0.97 1.21] | 0.99 [0.92 1.05] | 1.00 [0.92 1.09] | 0.98 [0.94 1.02] | 1.01 [0.94 1.09] | 1.01 [0.98 1.05] |
| **Proctectomy** *(ref = Unchanged volume)* | |  |  |  |  |  |
| *Increased* | 0.99 [0.82 1.19] | 0.95 [0.85 1.05] | 0.92 [0.79 1.06] | 0.98 [0.94 1.03] | 0.99 [0.91 1.08] | 0.97 [0.93 1.02] |
| *Decreased* | 0.96 [0.79 1.16] | 1.05 [0.92 1.20] | 0.91 [0.78 1.06] | 0.99 [0.93 1.05] | 0.95 [0.86 1.04] | 1.00 [0.94 1.05] |
| **Esophagectomy** *(ref = Unchanged volume)* | |  |  |  |  |  |
| *Increased* | 0.69 [0.35 1.36] | 0.65 [0.51 0.83] | 0.70 [0.45 1.09] | 0.79 [0.60 1.05] | 1.56 [1.08 2.25] | 1.01 [0.80 1.28] |
| *Decreased* | 0.92 [0.62 1.36] | 0.97 [0.75 1.26] | 0.94 [0.72 1.24] | 0.96 [0.84 1.10] | 1.16 [0.90 1.49] | 1.04 [0.91 1.20] |
| **Gastrectomy** *(ref = Unchanged volume)* | |  |  |  |  |  |
| *Increased* | 0.91 [0.66 1.25] | 1.01 [0.81 1.25] | 0.89 [0.73 1.08] | 0.96 [0.82 1.11] | 0.95 [0.83 1.10] | 1.02 [0.90 1.15] |
| *Decreased* | 1.11 [0.89 1.39] | 1.00 [0.88 1.15] | 0.99 [0.86 1.14] | 1.04 [0.95 1.13] | 0.98 [0.87 1.10] | 1.01 [0.94 1.10] |
| **Pancreatectomy** *(ref = Unchanged volume)* | |  |  |  |  |  |
| *Increased* | 1.39 [1.02 1.90] | 1.09 [0.90 1.32] | 0.91 [0.73 1.12] | 1.10 [0.98 1.23] | 0.97 [0.82 1.14] | 1.08 [0.98 1.19] |
| *Decreased* | 1.47 [0.95 2.27] | 0.95 [0.78 1.16] | 1.19 [0.96 1.46] | 0.93 [0.84 1.03] | 1.07 [0.90 1.28] | 0.92 [0.82 1.03] |
| **PCI** *(ref = Unchanged volume)* |  |  |  |  |  |  |
| *Increased* | 1.05 [0.90 1.21] | 0.97 [0.89 1.05] | 1.08 [0.90 1.30] | 1.05 [1.00 1.11] | 1.00 [0.85 1.17] | 0.98 [0.91 1.05] |
| *Decreased* | 1.13 [0.92 1.40] | 1.04 [0.95 1.13] | 0.89 [0.72 1.09] | 0.98 [0.94 1.03] | 0.89 [0.75 1.06] | 1.01 [0.97 1.05] |
| **CABG** *(ref = Unchanged volume)* |  |  |  |  |  |  |
| *Increased* | 0.75 [0.48 1.17] | 1.01 [0.81 1.26] | 0.71 [0.53 0.94] | 0.71 [0.53 0.94] | 1.06 [0.91 1.22] | 1.06 [0.91 1.22] |
| *Decreased* | 1.16 [0.59 2.28] | 1.03 [0.82 1.30] | 0.43 [0.22 0.82] | 1.53 [1.30 1.81] | 1.53 [1.30 1.81] | 1.06 [0.96 1.17] |
| **AAA repair** *(ref = Unchanged volume)* | |  |  |  |  |  |
| *Increased* | 0.92 [0.65 1.32] | 1.11 [0.86 1.45] | 1.08 [0.87 1.35] | 0.93 [0.81 1.06] | 1.03 [0.87 1.23] | 1.02 [0.90 1.14] |
| *Decreased* | 0.98 [0.58 1.67] | 0.85 [0.61 1.18] | 1.24 [0.94 1.64] | 0.78 [0.64 0.96] | 1.08 [0.89 1.31] | 0.95 [0.85 1.07] |
| **Carotid endarterectomy** *(ref = Unchanged volume)* | |  |  |  |  |  |
| *Increased* | 1.23 [0.99 1.53] | 0.98 [0.86 1.11] | 0.96 [0.80 1.17] | 1.02 [0.95 1.10] | 1.13 [1.01 1.27] | 0.99 [0.93 1.04] |
| *Decreased* | 1.19 [0.81 1.74] | 0.97 [0.78 1.22] | 1.31 [1.07 1.61] | 1.04 [0.91 1.20] | 1.34 [1.16 1.56] | 1.01 [0.93 1.09] |
| **Hip fracture repair** *(ref = Unchanged volume)* | |  |  |  |  |  |
| *Increased* | 1.05 [0.93 1.18] | 1.01 [0.95 1.07] | 0.97 [0.86 1.09] | 1.01 [0.97 1.07] | 1.01 [0.92 1.11] | 1.01 [0.97 1.05] |
| *Decreased* | 1.00 [0.88 1.13] | 0.96 [0.91 1.01] | 0.95 [0.88 1.03] | 1.00 [0.96 1.05] | 0.97 [0.90 1.04] | 1.00 [0.96 1.04] |
